# Efficacy and safety analysis of transarterial chemoembolization combined with tyrosine kinase inhibitors and immune checkpoint inhibitors with or without microwave ablation for unresectable hepatocellular carcinoma: a retrospective, multicenter, case-control study

**DOI:** 10.3389/fimmu.2025.1639515

**Published:** 2025-09-17

**Authors:** Nan Wang, Jingwen Xu, Chengxu Zhan, Pikun Cao, Tianyu Xue, Jinpeng Li, Zhigang Wei, Xin Ye

**Affiliations:** ^1^ Department of First Clinical Medical College, Shandong University of Traditional Chinese Medicine, Shandong Provincial Qianfoshan Hospital, Jinan, Shandong, China; ^2^ Department of Cardiology, The First Affiliated Hospital of Shandong First Medical University & Shandong Provincial Qianfoshan Hospital, Shandong Medicine and Health Key Laboratory of Cardiac Electrophysiology and Arrhythmia, Jinan, China; ^3^ Department of Oncology, The First Affiliated Hospital of Shandong First Medical University & Shandong Provincial Qianfoshan Hospital, Shandong Lung Cancer Institute, Jinan, China; ^4^ Department of Interventional Therapy I, Shandong Cancer Hospital and Institute, Shandong First Medical University and Shandong Academy of Medical Sciences, Jinan, Shandong, China

**Keywords:** hepatocellular carcinoma, microwave ablation, transarterial chemoembolization, immune checkpoint inhibitors, combined modality therapy

## Abstract

**Objective:**

To evaluate the efficacy and safety of transarterial chemoembolization (TACE) combined with tyrosine kinase inhibitors (TKIs) and immune checkpoint inhibitors (ICIs) with or without microwave ablation (MWA) for unresectable hepatocellular carcinoma (uHCC).

**Materials and methods:**

This retrospective study comprised 220 patients with uHCC who underwent TACE combined with a TKI and ICI with MWA (Group A: 105 patients (median age, 60 ± 10 years) and 82 (78.1%) were men) or without MWA (Group B: 115 patients (median age, 58.35 ± 10.27 years) and 97 (84.4%) were men) at multiple centers in China. The overall survival (OS), progression-free survival (PFS), objective response rate (ORR), and safety were compared between the two groups.

**Results:**

The OS, PFS, and ORR in Group A were significantly higher than those in Group B (OS, 21.30 ± 8.25 vs. 15.49 ± 7.41 months, *p* < 0.0001; PFS, 14.29 ± 6.34 vs. 7.15 ± 4.53 months, *p* < 0.0001; ORR, 66.7% [70/105] vs. 31.3% [36/115], *p* < 0.0001). The multivariable Cox regression analysis revealed that the combination of MWA and a more favorable tumor response were significantly associated with improved OS (hazard ratio, 0.5261; 95% confidence interval, 0.3839–0.7182; *p* = 0.0005 and hazard ratio, 0.5770; 95% confidence interval, 0.4209–0.7886; *p* = 0.0016). Grade 3 or 4 adverse events occurred in 30/105 (28.6%) and 29/115 (25.2%) patients in Groups A and B, respectively.

**Conclusion:**

The combination therapy (TACE + TKIs and ICIs) with MWA showed higher safety and significantly better OS, PFS, and ORR for uHCC than that without MWA.

## Introduction

Hepatocellular carcinoma (HCC) accounts for 75%–90% of all liver cancer cases and is ranked as the sixth most common malignancy and third leading cause of cancer-related mortality worldwide (1). Despite efforts to prevent and manage this disease, the burden of HCC continues to increase, and the number of newly diagnosed HCC cases annually is predicted to reach over 1 million by 2025 (2–4). The treatment options for early to middle-stage HCC comprise local therapy, such as liver transplantation, surgery, ablation, or transarterial chemoembolization (TACE) (5–7). Unfortunately, due to the unique biological characteristics of HCC, most HCC cases are first identified when the disease has reached an unresectable stage (8, 9). Consequently, it is of paramount importance to devise a therapeutic strategy aimed at enhancing the survival prospects for patients with unresectable HCC (uHCC). Local therapy without systemic therapy poses many challenges, such as local recurrence, distant metastasis, and multiple tumors, during the treatment of uHCC (10, 11). Recently, the combination of tyrosine kinase inhibitors (TKIs) or bevacizumab and immune checkpoint inhibitors (ICIs) has been used as first-line treatment for patients with uHCC (12, 13). However, systemic therapy is associated with several problems, such as an insufficient response rate, concomitant toxicity, and drug resistance (14–16). Several recent studies and clinical trials have preliminarily reported the efficacy of TACE combined with system therapy in uHCC, thereby demonstrating the therapeutic value of combined local and systemic therapy (17–20).

The advantages of microwave ablation (MWA), a thermal ablation technique using electromagnetic waves, include a faster ablation process, wider ablation zone, less heat sink, and enhanced immune response, which is suitable for treating HCC (21–23). Previous studies have reported that TACE plus MWA can improve the tumor necrosis rate and the median overall survival (OS) in patients with uHCC (24–26). Theoretically, MWA could play an important role in the combination treatment strategy for uHCC. Therefore, this study aimed to evaluate the efficacy and safety of TACE plus MWA combined with a TKI and ICIs in patients with uHCC.

## Materials and methods

### Patients

**Figure 1:**
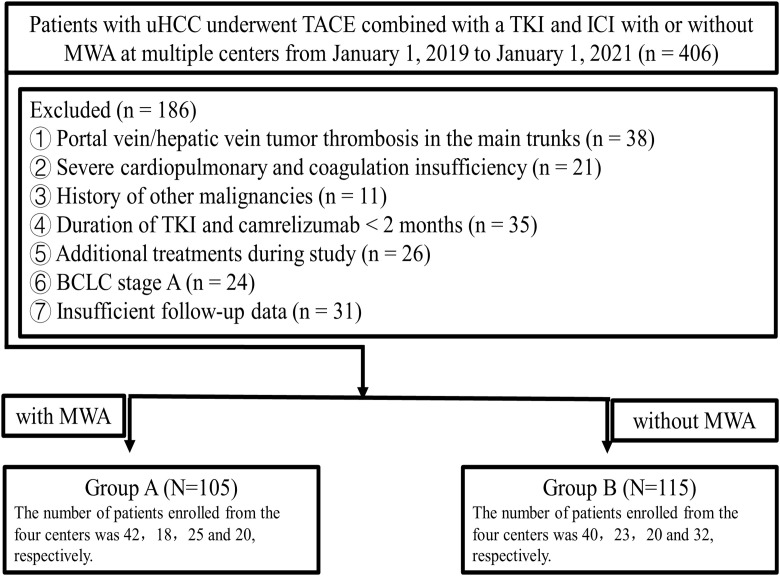
Flowchart of the patient selection process. uHCC, unresectable hepatic carcinoma; TACE, transarterial chemoembolization; TKI, tyrosine kinase inhibitor; ICIs, Immune checkpoint inhibitors; MWA, microwave ablation; BCLC, Barcelona Clinic Liver Cancer.

This retrospective study collected data between January 1, 2019 and January 1, 2021, from four medical centers (The First Affiliated Hospital of Shandong First Medical University, Jinan, Shandong, China; Affiliated Hospital of Shandong University of Traditional Chinese Medicine, Jinan, Shandong, China; Shandong Provincial Hospital Affiliated to Shandong First Medical University, Jinan, Shandong, China; Affiliated Cancer Hospital of Shandong First Medical University, Jinan, Shandong, China). Approval for this retrospective study, which complied with the standards of the Declaration of Helsinki, was obtained from Institutional Ethics Committee of the four relevant participating institutions, and informed consent was waived due to the retrospective nature of this study. The diagnosis of HCC was based on the guidelines of the European Association for the Study of the Liver and the American Association for the Study of Liver Diseases. The participant selection process is depicted in the flowchart in **Figure 1**. A total of 220 patients were included in this study (105 patients (median age, 60 ± 10 years) and 82 (78.1%) were men in Group A who underwent TACE plus MWA combined with a TKI and ICI and 115 patients (median age, 58.35 ± 10.27 years) and 97 (84.4%) were men in Group B who underwent TACE combined with a TKI and ICI.

The treatment decisions are based on the patient’s circumstances and the physician’s discretion. Multidisciplinary teams at the participating hospitals, which specialize in HCC, primarily make treatment decisions in accordance with the Barcelona Clinic Liver Cancer (BCLC) guidelines or the China National Liver Cancer guidelines. Before reaching a final decision, physicians ensure that patients and their family members are informed of the benefits and drawbacks of various treatment protocols, including potential treatment outcomes, financial implications, and treatment-related complications.

The inclusion criteria in this study were as follows: eligible patients ≥18 years old with uHCC and at least one measurable lesion according to Response Evaluation Criteria in Solid Tumors version 1.1; an Eastern Cooperative Oncology Group performance status (ECOG PS) of ≤ 2; a Child–Pugh classification of A–B; and no coagulation dysfunction. Patients received the combination therapy (TACE + TKIs and ICIs) with or without MWA during the same period. Those with the following conditions were excluded: portal vein/hepatic vein tumor thrombosis in the main trunks; history of encephalopathy or refractory ascites; severe cardiopulmonary and coagulation insufficiency; history of other malignant tumors; existing immunodeficiency disease or a history of organ transplantation; duration of TKI and ICIs <2 months; Surgically respectable HCC; incomplete clinical or follow-up data. Follow-up was terminated on January 1, 2023.

### Treatment sequencing

The patients were divided into two groups: Group A underwent TACE plus MWA combined with a TKI and ICIs, and Group B underwent TACE combined with a TKI and ICIs.

#### TKIs

TKIs (initiated 3-5 days pre-TACE): To normalize tumor vasculature and enhance subsequent chemoembolization delivery. The TKIs mainly included sorafenib (400 mg twice a day), lenvatinib (8 or 12 mg once a day, based on body weight), or apatinib (250 mg once a day), and dose reduction for TKIs due to toxicity was allowed. One of these medicines was selected based on the willingness and tolerance of the patient.

#### TACE

The patients included in the study received conventional TACE (cTACE) or drug-eluting beads TACE (DEB-TACE), which were performed according to a standard protocol by interventional radiologists from the participating centers with at least 10 years of experience. The treatment strategy for TACE was done “on-demand,” meaning it was implemented when incomplete necrosis of new lesions was suspected in the intrahepatic tumor. Subsequent TACE was discontinued when the patient’s liver function deteriorated to Child-Pugh class C (uncontrollable ascites, severe jaundice, overt hepatic encephalopathy, or hepatorenal syndrome), the ECOG performance status was >2, or the target lesions continuously progressed after three TACE sessions. cTACE was performed by injecting chemotherapy medicines and embolic agents into the tumor-feeding artery. The chemotherapeutic regimens used were determined by a multidisciplinary team (MDT) with standardized training prior to the procedure. Chemotherapy medicines like doxorubicin, epirubicin, and platinum were commonly used. The size and type of the embolic agent were determined according to the clinical practice of the participating center. In the case of DEB-TACE, various sizes (100–300and 300–500 um) of the embolic agent callispheres (Suzhou Hengrui Callisyn Biomedical Co.) loaded with 60 mg of doxorubicin per vial were used. The endpoints of embolization were the complete disappearance of the vessels supplying blood to the tumor or the development of the main trunk only and the gradual reduction in the flow of the contrast agent followed by its disappearance after 2–5 heartbeats. In general, the embolization endpoint of DEB-TACE is required to reach “near stasis,” the endpoint of cTACE should reach “complete stasis,” during the super-selective catheterization of the tumor-feeding artery (27).

#### ICIs

ICIs (administered 3-7 days post-TACE): To capitalize on tumor antigen release from chemoembolization-induced necrosis. Patients with Child–Pugh class A and B would receive the ICI (pembrolizumab, nivolumab, sintilizumab, and camrelizumab). ICI therapy was suggested at a standard dose once every 3 weeks. Temporary ICI interruption was allowed due to toxicity, but dose reduction was not allowed.

#### MWA

The MDT composed of hepatobiliary surgeons, radiation oncologists, medical oncologists, and interventional radiologists conducted the case assessment. The indication for MWA was determined primarily by the identification of residual tumor following TACE, rather than by specific patient baseline characteristics. Additionally, procedural feasibility—defined by the availability of a safe and accessible ablation pathway as confirmed by medical imaging examination data—constituted an essential technical prerequisite for treatment allocation. A physician with more than 15 years of experience in minimally invasive treatment and a physician with more than 10 years of experience in imaging worked together to complete the preprocedural plan. The MWA performers in this study were experienced through unified training and clinical cases and adhered to the MWA technical guidelines for treating hepatic tumors. The ECO-100 water-cooled microwave device (ECO Microwave Electronic Institute, Nanjing, China) and microwave antenna (19G) were used for MWA. After routine preparation, a plain computed tomography (CT; Somatom 64 Sensation; Siemens, Muenchen, Germany) scan was performed to confirm the target tumor and puncture the path according to the preprocedural plan. Typically, the selection of the MWA strategy and the target tumor during preprocedural planning was based on the following criteria: a multidisciplinary consultation, wherein the target lesions were deemed resistant to regular on-demand TACE combined with systemic therapy; the presence of less than three new lesions while undergoing MWA; and the ability to deactivate all the target lesions during a single MWA procedure. A single antenna was generally used for the complete ablation of tumors with a maximum diameter of <30 mm. Alternatively, multiple overlapping ablations were performed to accurately judge the required number of ablations for tumors with a maximum diameter of >30 mm. The location of the needle placement effectively reduced residual tumors or recurrence. The treatment parameters were set at 50–70 watts, and the procedure lasted 7–15 min. After the MWA procedure, an immediate CT scan was performed to assess the ablation zone and the presence of complications.

##### Antiviral treatment for hepatitis B virus

Antiviral treatment regimens were determined by hepatology specialists within the MDT. Patients underwent regular follow-up and virological monitoring. HBV DNA quantification was not performed as part of routine surveillance.

### Assessments

All patients were required to undergo a follow-up with laboratory tests and enhanced CT/Nuclear Magnetic Resonance Imaging (MRI) scans at intervals of 4–6 weeks after the first treatment. Tumor response was evaluated every 4–6 weeks after each treatment. The responses, including complete response (CR), partial response (PR), stable disease (SD), and progressive disease (PD), were evaluated by two radiologists, both with >10 years’ experience, using the modified Response Evaluation Criteria in Solid Tumors (mRECIST) and compared between the two groups. The objective response rate (ORR), disease control rate (DCR), and duration of response (DOR) were also assessed. OS was defined as the period from the date of uHCC diagnosis until the last follow-up date (or death). Progression-free survival (PFS) was defined as the interval between the initial TACE and radiologic disease progression (or death). Those who remained alive at the last follow-up date were considered “censored.”

### Follow-up and management of adverse events

Patients with no tumor progression were advised to undergo follow-up every 3 months. Repeated MWA or TACE for residual tumors required a consensus decision made by the multidisciplinary team. The timing of subsequent TACE or MWA procedures depended on the treatment efficacy and the patient’s recovery. The severity of the AEs was assessed according to the Common Terminology Criteria for Adverse Events (CTCAE - Version 5.0). Grade 3 or 4 TKI-related AEs were encountered during targeted therapy, the medicine dose was reduced until the toxicity was manageable (28). In case of unmanageable ICI-related AEs, cancellation of the immunotherapy was recommended. Ideally, continuous treatment with TKIs and ICIs was recommended when TACE or MWA was performed unless unmanageable toxicity or uncontrolled disease progression was encountered.

### Statistical analysis

SPSS version 22.0 (IBM Corporation, Armonk, NY, USA) was used for the statistical analysis. Quantitative data are expressed as frequency, mean ± standard deviation, or median, with a 95% confidence interval (CI). The Mann-Whitney U test (non-normally distributed data) or Student’s t-test (normally distributed data) was performed for continuous variables analysis, and the Chi-square or Fisher’s exact test was employed to compare categorical variables. Survival curves for PFS and OS were estimated using the Kaplan–Meier method. Univariate analyses were performed to identify the predictive factors of survival using the log-rank test. Variables with a *p*-value of <0.1 in the univariate analysis were used for the multivariate analysis using the Cox proportional hazard regression model. For all tests, a *p*-value of <0.05 was considered statistically significant. Subgroup analysis comparing the PFS and OS between the two groups was performed for pre-specified clinically relevant parameters.

## Results

### Demographic and treatment characteristics

No significant differences in basic characteristics, including age, sex, ECOG score, BCLC stage, size of the tumors (cm), HBsAg positive, Child-Pugh class, alpha-fetoprotein (AFP) level, and albumin-bilirubin (ALBI) grade were observed between the two groups. Likewise, there were also no statistically significant differences between the two groups in the type of TACE treatment, the type of TKIs, and the type of ICIs. The institutional distribution is presented in **Table 1**.

**Table 1:** Baseline characteristics of the patients.

Characteristic*	Group A (N = 105)	Group B (N = 115)	P-value
Age, years (range)^#^	60 ± 10 (36-80)	58.35 ± 10.27 (24-80)	0.1654
Sex			0.7048
Male	82 (78.1)	97 (84.3)	
Female	23 (21.9)	18 (15.7)	
ECOG PS			0.5577
0	40 (38.1)	49 (42.6)	
1	56 (53.3)	54 (46.9)	
2	9 (8.6)	12 (10.5)	
CNLC stage			0.4045
2B	8 (7.6)	6 (5.2)	
3A	52 (49.5)	61 (53.1)	
3B	45 (42.9)	48 (41.7)	
BCLC stage			0.6051
B	8 (7.6)	6 (5.2)	
C	97 (92.4)	109 (94.8)	
HBsAg positive	40 (38.1)	53 (46.1)	0.6444
Child–Pugh class			0.8834
A	84 (80.0)	62 (53.9)	
B	21 (20.0)	53 (46.1)	
AFP (ng/mL)			0.5598
<400	39 (37.1)	45 (39.1)	
≥400	29 (27.6)	39 (33.9)	
N.A.	37 (35.3)	31 (27.0)	
ALBI grade			0.3440
1	37 (35.2)	39 (33.9)	
2	68 (64.8)	76 (66.1)	
Vascular invasion			0.7284
Yes	73 (69.5)	89 (77.4)	
No	32 (30.5)	26 (22.6)	
Tumor diameter, cm (range)	9.69 ± 4.72 (2 - 26)	9.86 ± 5.00 (2 - 22)	0.7942
Tumor number			0.2422
Single	18 (17.1)	25 (21.7)	
Multiple	87 (82.9)	90 (78.3)	
Extrahepatic metastasis			0.5502
Yes	45 (42.9)	48 (41.7)	
No	60 (57.1)	67 (58.3)	
Types of TACE procedure			0.3755
cTACE	219 (69.5)	436 (75.8)	
DEB-TACE	96 (30.5)	139 (24.2)	
Types of TKIs			0.4168
Sorafenib	21 (20.0)	29 (25.2)	
Lenvatinib	17 (16.2)	14 (12.2)	
Apatinib	67 (63.8)	72 (62.6)	
Types of ICIs			0.5809
Pembrolizumab	15 (14.3)	14 (12.2)	
Nivolumab	7 (6.7)	11 (9.6)	
Sintilizumab	28 (26.7)	22 (19.1)	
Camrelizumab	55 (52.3)	68 (59.1)	

*Except where indicated, data are number (%). The Chi-square or Fisher exact test was applied for categorical variables. # Data were continuous variables, expressed in average ± standard deviation (range), and were compared using the Mann-Whitney U test. Among HBsAg-positive patients, 89% received antiviral therapy with entecavir or tenofovir.

ECOG PS, Eastern Cooperative Oncology Group performance status; CNLC, China liver cancer staging; BCLC, Barcelona Clinic Liver Cancer; AFP, a-fetoprotein; N/A, not applicable; ALBI, Albumin-Bilirubin; cTACE, conventional transarterial chemoembolization; DEB-TACE, drug-eluting beads transarterial chemoembolization; TKIs, Tyrosine Kinase Inhibitors; ICIs, Immune checkpoint inhibitors.

### Efficacy and survival

**Figure 2:**
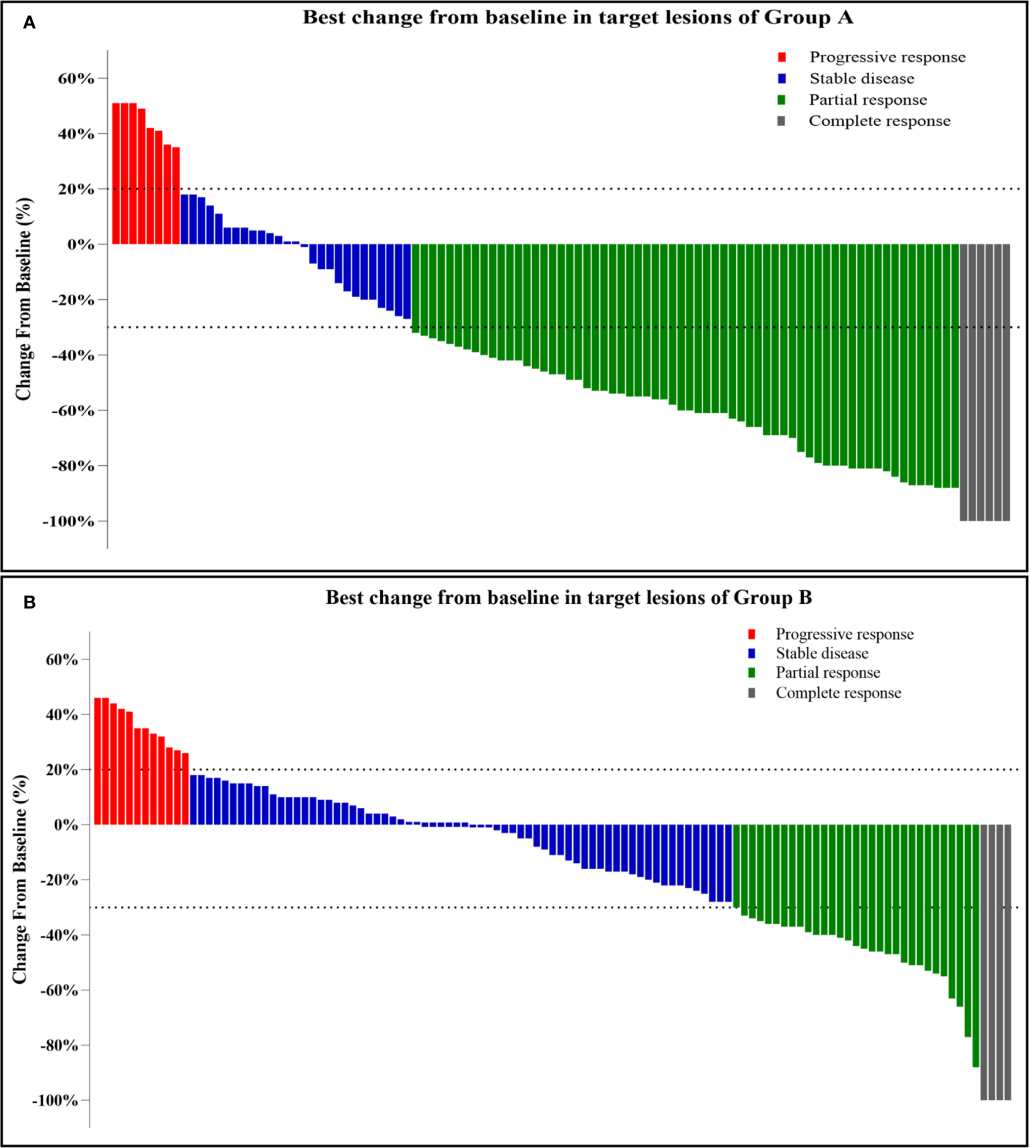
**(A, B)** Best change from baseline in the sum of the target lesion diameter per patient. Tumor response was assessed by mRECIST: Progressive response (≥20% increase or new lesions); Stable disease (no qualifying shrinkage or growth); Partial response (≥30% decrease in viable tumor diameter); Complete response (disappearance of enhancing lesions).

**Table 2** presents the results of the efficacy and survival in the two groups. As of January 1, 2023, the median follow-up duration was 23.0 ± 11.2 months (range, 3–45 months) in Group A and 19.7 ± 10.6 months (range, 3–37 months) in Group B. The median number of sessions of TACE were 2.98 ± 1.73 (range: 1–12) in Group A and 5.04 ± 2.35 (range: 1–17) in Group B, respectively. The median number of MWA treatment sessions was 2.17 ± 1.34 (range: 1–7) in Group A. According to mRECIST, patients in Group A achieved a higher ORR, DCR, and DOR than those in Group B [70/105 (66.7%) vs. 36/115 (31.3%), *p* < 0.0001; 97/105(92.4%) vs. 103/115 (89.6%), *p* < 0.0001, and 9.92 ± 6.62 (0.51–29.9) vs. 4.90 ± 4.45 (0.37–23.40) months, respectively]. **Figure 2** showed the best change from baseline in the sum of the target lesion diameter per patient.

**Table 2:** Summary of the clinical efficacy of the treatment method in the two groups.

Characteristic*	All Patients (N = 220)	Group A (N = 105)	Group B (N = 115)	P-value
Objective response				<0.0001
CR	10 (4.5)	6 (5.7)	4 (3.5)	
PR	96 (43.6)	64 (60.9)	32 (27.8)	
SD	94 (42.7)	27 (25.8)	67 (58.3)	
PD	20 (9.2)	8 (7.6)	12 (10.4)	
ORR	106 (48.2)	70 (66.7)	36 (31.3)	<0.0001
DCR	200 (90.9)	97 (92.4)	103 (89.6)	0.4681
TTR, (range) ^#^	2.46 ± 0.79 (1.01-4.07)	2.18 ± 0.73 (1.01-3.57)	2.71 ± 0.76 (1.41-4.07)	<0.0001
DOR, (range)	7.30 ± 6.12 (0.37-29.9)	9.92 ± 6.62 (0.51-29.9)	4.90 ± 4.45 (0.37-23.40)	<0.0001
Follow-up (range)	21.13 ± 10.9 (3–45)	23.0 ± 11.2 (3–45)	19.7 ± 10.6 (3–37)	
PFS	10.55 ± 6.52	14.29 ± 6.34	7.15 ± 4.53	<0.0001
OS	18.26 ± 8.33	21.30 ± 8.25	15.49 ± 7.41	<0.0001

*Except where indicated, data are number (%). The Chi-square or Fisher exact test was used for categorical variables.

#Data were continuous variables, expressed in average ± standard deviation (range), and were compared using the Mann–Whitney U test.

CR, complete response; PR, partial response; SD, stable disease; PD, progressive disease; ORR, overall response rate; DCR, disease control rate; TTR, Time to response; DOR, Duration of response; PFS, progression-free survival; OS, overall survival.

**Figure 3:**
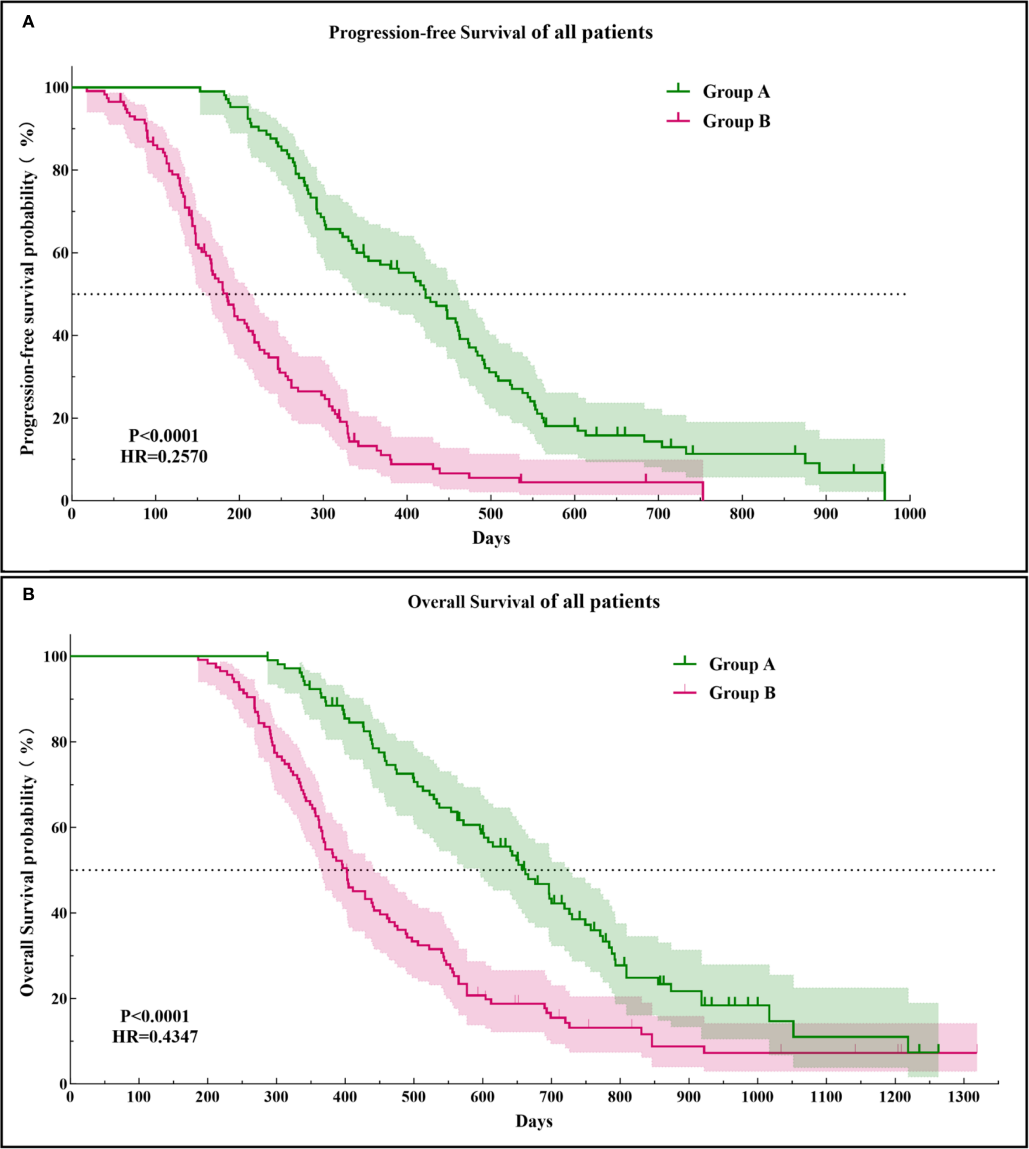
Kaplan–Meier curves of the progression-free survival **(A)** and overall survival **(B)** in both groups of patients. HR, hazard ratio.

**Figure 4:**
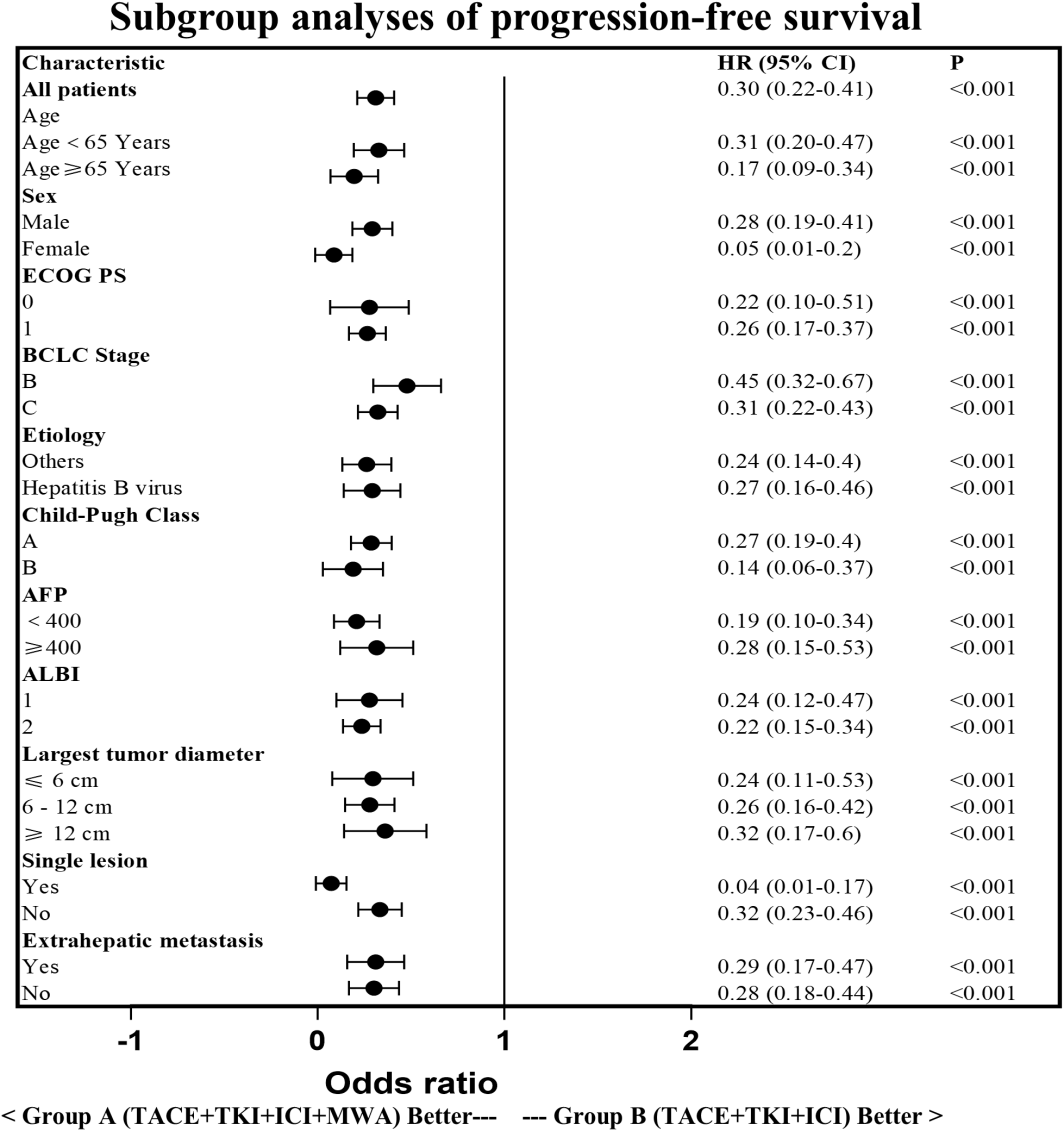
Subgroup analysis of the progression-free survival. HR, hazard ratio; CI, confidence interval; ECOG,Eastern Cooperative Oncology Group; BCLC, Barcelona Clinic Liver Cancer; AFP, a-fetoprotein; ALBI, Albumin-Bilirubin.

**Figure 5:**
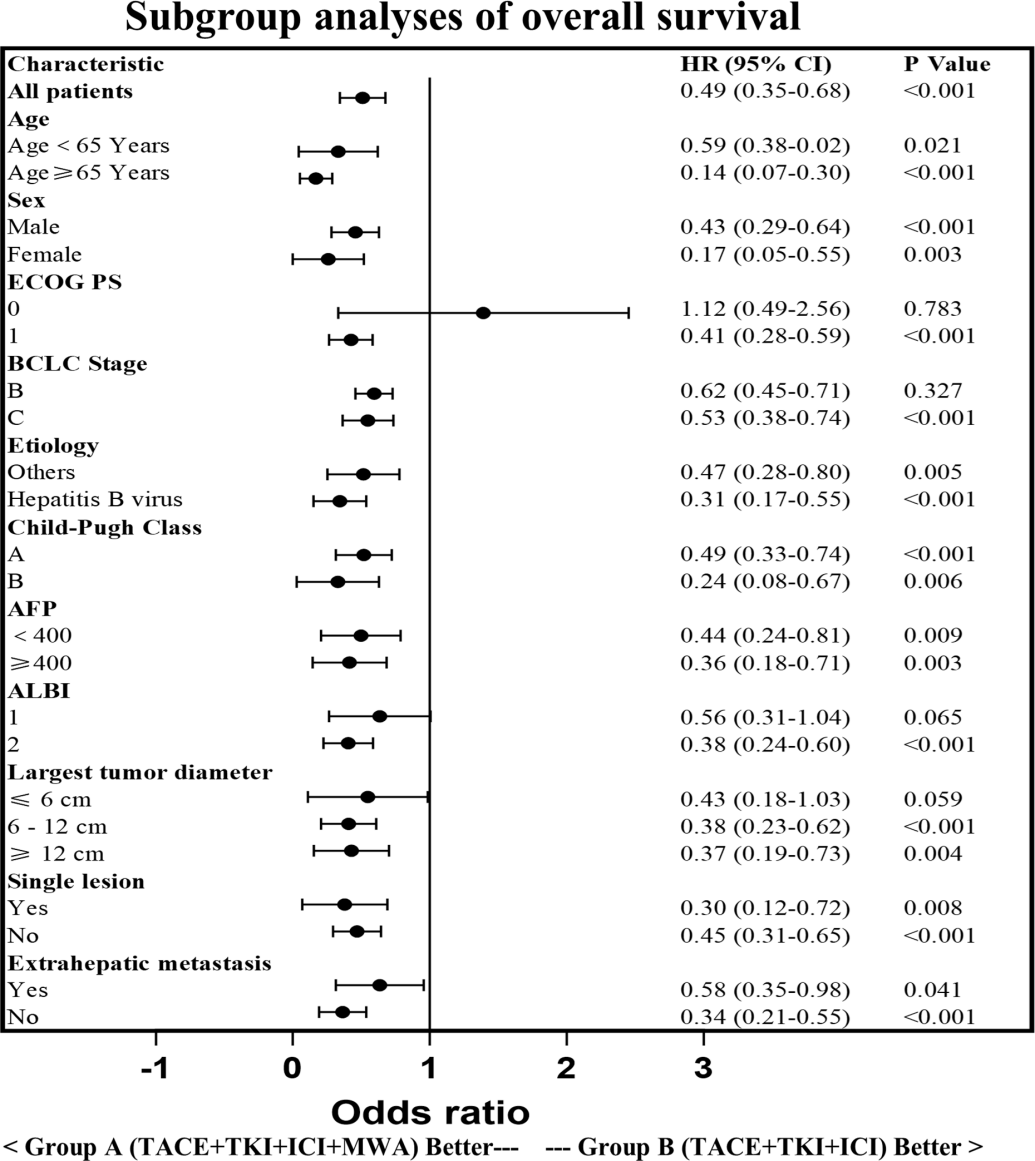
Subgroup analysis of the overall survival. HR, hazard ratio; CI, confidence interval; ECOG,Eastern Cooperative Oncology Group; BCLC, Barcelona Clinic Liver Cancer; AFP, a-fetoprotein; ALBI, Albumin-Bilirubin.

The PFS in Group A was significantly higher than that in Group B (14.29 ± 6.34 vs. 7.15 ± 4.53 months, *p* < 0.0001). Similarly, the OS in Group A was significantly higher than that in Group B (21.30 ± 8.25 vs. 15.49 ± 7.41 months, *p* < 0.0001; **Figure 3**). Subgroup analysis showed a trend that persisted in longer PFS and OS benefits with Group A compared to Group B (**Figures 4**, **5**).

### Prognostic factors analysis of OS

**Table 3** shows the results of the univariate and multivariate analyses of the prognostic factors for OS. The univariate analyses revealed that ECOG PS, BCLC stage, Hepatitis B virus, diameter of lesions, number of lesions, metastasis, tumor response, and MWA treatment were significantly associated with OS. In the multivariate analysis, tumor response (CR + PR vs. SD+PD: HR = 0.5770; 95% CI = 0.4209–0.7886; *p* = 0.0016) and MWA treatment (with MWA vs. without MWA: HR = 0.5261; 95% CI = 0.3839–0.7182; *p* = 0.0005) were identified as independent prognostic factors for OS.

**Table 3:** Univariate and multivariate analyses of the prognostic factors for overall survival.

Characteristic	Univariable Analysis			Multivariable Analysis		
	HR	95% CI	P value	HR	95% CI	P value
Age, years (<65 vs. ≥65)	0.6361	0.3341–1.211	0.1686			
Sex (male vs. female)	1.41	0.8693–2.286	0.1638			
ECOG PS (0 vs. 1 and 2)	0.5734	0.4102–0.8016	0.0011	0.8811	0.6523–1.1854	0.9570
BCLC stage (B vs. C)	0.4933	0.3695–0.6585	<0.0001	0.7273	0.3389–1.3724	0.6191
Hepatitis B virus (yes vs. no)	1.973	1.468–2.653	<0.0001	1.3493	0.8182–2.1259	0.8782
Child-Pugh class (A vs. B)	0.5396	0.3034–0.9596	0.1857			
AFP level (≥400 vs.<400 ng/mL)	1.113	0.739–2.032	0.107			
ALBI grade (1 vs. 2)	0.7927	0.5484–0.9067	0.2276			
Tumor diameter (<9 vs. ≥9 cm)	0.4702	0.3202–0.6905	0.0001	0.993	0.7305–1.3463	0.2059
Single lesion (yes vs. no)	0.54	0.3953–0.7376	0.0001	0.9252	0.6489–1.349	0.2896
Metastasis (yes vs no)	1.949	1.270–2.991	0.0023	1.187	0.8770–1.602	0.2625
Tumor response (CR + PR vs. SD + PD)	0.2794	0.1910–0.4088	<0.0001	0.577	0.4209–0.7886	0.0016
Combined with MWA (yes vs. no)	0.2175	0.1438–0.3291	<0.0001	0.5261	0.3839–0.7182	0.0005

The multivariable analysis includes the variables with p-value ≤0.1 from the univariable analysis.

HR, hazard ratio; CI, confidence intervals; ECOG PS, Eastern Cooperative Oncology Group performance status; BCLC, Barcelona Clinic Liver Cancer; cm, centimeter; AFP, a-fetoprotein; ALBI, Albumin–Bilirubin; CR, complete response; PR, partial response; SD, stable disease; PD, progressive disease; MWA, Microwave ablation.

### Systemic therapeutic agents

**Table 4** presents the types and proportions of systemic therapeutic agents utilized in the two groups. Lenvatinib, sorafenib, and apatinib were administered to 14.09%, 22.73%, and 63.18% of patients, respectively. Pembrolizumab, nivolumab, sintilimab, and camrelizumab were used in 13.18%, 8.18%, 22.73%, and 55.91% of patients, respectively. In terms of combination therapy, the regimen of apatinib plus camrelizumab was the most common, accounting for 48.64% of cases, while all other combinations each constituted less than 10%. Univariate analysis of the impact of each TKI or ICI on OS showed that OS advantage was largely consistent between the two groups.

**Table 4:** Types and proportions of systemic therapeutic agents utilized in the two groups.

Types of TKIs	OS [days, Median (Q25, Q75)] of A group	OS [days, Median (Q25, Q75)] of B group	p value
Sorafenib	554 (426, 662)	343 (274, 396)	<0.0001
Lenvatinib	652 (473, 771)	345 (310.5, 394.25)	<0.0001
Apatinib	651 (458.5, 790)	489 (360, 648.25)	0.0033
Types of ICIs			
Pembrolizumab	596 (407, 722.5)	385 (290.5, 430.5)	0.0037
Nivolumab	652 (604.5, 740)	324 (295, 354)	<0.0001
Sintilizumab	498 (370.25, 625.5)	403 (353, 491.75)	0.1285
Camrelizumab	696 (521, 832)	446.5 (315.5, 620.75)	<0.0001
TKI+ICI			
Apatinib + Camrelizumab	698 (517, 857.25)	522 (362, 690)	0.0022
Sorafenib + Sintilizumab	554 (444, 656)	384 (360.5, 470)	0.0522
Lenvatinib + Sintilizumab	503 (397.25, 711.5)	402 (345, 416.5)	0.1004

Non-normally distributed variables are expressed as the median (Q25, Q75).

TKIs, tyrosine kinase inhibitors; ICIs, Immune checkpoint inhibitors; OS, overall survival.

### Safety

**Table 5** shows the incidence and severity of AEs associated with TACE, TKIs, ICIs, and MWA. A total of 69/105 (65.7%) patients in Group A and 84/115 (73.0%) patients in Group B experienced treatment-emergent AEs from TACE, TKIs, and ICIs. AEs associated with MWA occurred in 26/105 (24.8%) patients in Group A. Grade 3 or 4 AEs were observed in 30/105 (28.6%) patients in Group A and 29/115 (25.2%) patients in Group B. No treatment-related mortalities were observed in either group. Discontinuation of ICIs was documented in 7/105 (6.7%) patients in Group A and 10/115 (8.7%) patients in Group B. Similarly, TKI discontinuation due to AEs was observed in 8/105 (7.6%) patients in Group A and 10/115 (8.7%) patients in Group B. Dose interruptions of ICIs were reported in 9/105 (8.6%) patients in Group A and 11/115 (9.6%) patients in Group B, while dose reductions or interruptions of TKIs were experienced by 17/105 (16.2%) patients in Group A and 14/115 (12.2%) patients in Group B. The AEs mainly included abnormal transaminases, increased blood bilirubin, ascites, pain, anemia, proteinuria, nausea, hypertension, hand and foot syndrome, leukopenia, hypothyroidism, gastrointestinal hemorrhage immune-related pneumonia, rash, platelet count decrease, and weight loss. Furthermore, in a cohort comprising 55 patients from Group A and 68 patients from Group B, where camrelizumab was employed, the proportion of patients experiencing reactive cutaneous capillary endothelial proliferation (RCCEP) was 28/55 (50.9%) in Group A and 33/68 (48.5%) in Group B. RCCEP was not detected in patients who were treated with other ICIs. Antiviral therapy with entecavir or tenofovir was administered to 89% of HBsAg-positive patients. Pharmacy dispensing records indicated a medication adherence rate exceeding 90%. No instances of HBV reactivation were documented.

**Table 5:** Complications and adverse events related to TKI, camrelizumab, and TACE.

Adverse Events, n (%)	Group A (N = 105)	Group B (N = 115)
AEs (Exclude MWA related)	69 (65.7%)	84 (73.0%)
MWA related AEs	26 (24.8%)	N.A.
Grades 1 or 2 event*	65 (61.9%)	55 (47.8%)
Grades 3 or 4 event*	30 (28.6%)	29 (25.2%)
Grade 5 event	0	0
Discontinuation of ICIs#	7 (6.7%)	10 (8.7%)
Discontinuation of TKIs#	8 (7.6%)	10 (8.7%)
Dose interruption of ICIs	9 (8.6%)	11 (9.6%)
Dose reduction or interruption of TKIs	17 (16.2%)	14 (12.2%)
Abnormal transaminases	77 (73.3%)	71 (61.7%)
Increased blood bilirubin	46 (43.8%)	39 (33.9%)
Ascites	9 (8.6%)	16 (13.9%)
Pain	57 (54.3%)	40 (34.8%)
Anemia	13 (12.4%)	11 (9.6%)
Proteinuria	19 (18.1%)	26 (22.6%)
Nausea	61 (58.1%)	43 (37.4%)
Hypertension	67 (63.8%)	71 (61.7%)
Hand and foot syndrome	11 (10.5%)	16 (13.9%)
Leukopenia	24 (22.9%)	29 (25.2%)
Hypothyroidism	9 (8.6%)	17 (14.8%)
Gastrointestinal hemorrhage	4 (3.8%)	7 (6.1%)
Immune-related pneumonia	13 (12.4%)	10 (8.7%)
Rash	21 (20.0%)	19 (16.5%)
Platelet count decreased	49 (46.7%)	53 (46.1%)
Weight loss	17 (16.2%)	19 (16.5%)
RCCEP	28 (26.7%)	33 (28.7%)

Unless otherwise indicated, data are presented as the number of patients, with percentages in parentheses.

n, number of patients; AEs, adverse events; MWA, microwave ablation; ICIs, Immune checkpoint inhibitors; TKI, tyrosine kinase inhibitors; RCCEP, reactive cutaneous capillary endothelial proliferation.

## Discussion

Sorafenib has been used as the standard therapy for uHCC, with a low effective rate and a high drug resistance rate, for decades. After the IMbrave 150 clinical trial showed that bevacizumab combined with atezolizumab significantly improved the ORR, mOS, and mPFS compared to sorafenib alone, the Food and Drug Administration authorized this combination therapy (bevacizumab + atezolizumab) as the first-line treatment for uHCC in 2020 (29, 30). In addition, the KEYNOTE 524 (lenvatinib + pembrolizumab) and Orient 32 (sintilimab + bevacizumab analog) clinical trials demonstrated the improved efficacy of targeted therapy plus immunotherapy in uHCC (31, 32). Nonetheless, the overall effective rate of systemic therapy alone is not sufficient for patients with uHCC due to the significant tumor burden, multiple tumors, tumor heterogeneity, and development of portal vein thrombus.

TACE, which can quickly and accurately reduce the initial tumor burden, embolize most of the blood supply vessels, and effectively treat portal vein thrombus, is the primary treatment method for uHCC. The inclusion of TACE may overcome the drawbacks of targeted therapy plus immunotherapy and increase the overall efficacy of the treatment (33). Recent studies have demonstrated the safety and survival benefits of TACE in combination with targeted therapy and immunotherapy when compared to local or systemic therapy alone, which could improve the conversion resection rate of HCC. A recent clinical study (CHANCE2211) found that TACE plus camrelizumab and apatinib showed significantly better efficacy for predominantly advanced HCC than TACE monotherapy (34). The search for local therapies in combination with systemic therapy for the treatment of HCC is ongoing (35–37). Thermal ablation, particularly MWA, is a key local therapy for early-stage HCC, offering advantages such as larger ablation zones, higher temperatures, and reduced heat-sink effects (38, 39). TACE complements ablation by identifying occult tumors via arteriography and inducing ischemia that enlarges ablation volume. It may also mitigate hemorrhage and seeding risks from MWA, while MWA targets residual tumors post-TACE (40–42). This study demonstrates that adding MWA to TACE plus systemic therapy (TKI and ICI) improved outcomes. Group A (with MWA) showed significantly superior PFS and OS versus Group B. Although non-randomized allocation may introduce selection bias, the decision for MWA was technically driven (targeting TACE residuals), minimizing bias. The survival benefit likely stems from MWA’s biological effects—TME modulation, immunologic synergy, and TACE session reduction preserving liver function—rather than baseline differences. Group A also achieved higher CR/PR rates (**Figure 2**), attributable to synergistic tumor inactivation, enhanced antigen release, and possible conversion to immunogenic “hot” tumors promoting ICI response. Group B outcomes (PFS 7.15 ± 4.53 months, OS 15.49 ± 7.41 months, ORR 31.3%) were lower than in CHANCE2211, likely due to more advanced disease and higher tumor burden. AE profiles were comparable between groups. Over 90% received guideline-based antiviral therapy. While HBV positivity influenced OS in univariate analysis (p < 0.0001), it was not independent on multivariate testing (p = 0.8782). Previous studies indicate that combining antiviral therapy with TACE/RFA improves liver function and survival in advanced HCC (43). Heterogeneity in HBV DNA quantification across centers precluded incorporating viral load; its inclusion might have refined prognostic accuracy.

Combined modality therapy involving TACE, MWA, TKIs, and ICIs remains relatively underexplored in HCC, despite growing interest in integrating local and systemic treatments for uHCC (44, 45). MWA following TACE enables precise tumor destruction and enhances tumor response, while jointly promoting the release of tumor antigens and stimulating anti-tumor immunity (46). However, TACE-induced hypoxia elevates Vascular Endothelial Growth Factor (VEGF) expression, fostering an immunosuppressive and pro-metastatic tumor microenvironment. TKIs counter this by blocking VEGF signaling, inhibiting aberrant angiogenesis, and promoting vascular normalization, thereby enhancing drug delivery and T-cell infiltration (47). ICIs further augment anti-tumor immunity by facilitating immune recognition of tumor cells. Evidence indicates MWA rapidly enhances antigen presentation, activates innate and adaptive immunity, and reverses immunosuppression, albeit transiently (4–7 days) (48, 49). Combined MWA and immunotherapy amplifies CD8+ T-cell function and memory formation, enriches migratory type 1 dendritic cells, and expands tumor-specific T cells, sustaining immune activity in the TME (50, 51). TKIs synergize by sustaining immune activation through vascular normalization and ameliorating VEGF-mediated immunosuppression (52). Owing to the retrospective design, pre-treatment tissue was unavailable and biomarker analysis (e.g., Programmed Death-Ligand 1, Tumor Mutational Burden) was unstandardized across centers, limiting correlative biological insights. Nonetheless, the improved ORR and PFS in the MWA group suggest therapeutic synergy, underscoring the need for prospective trials with integrated biomarker profiling. Although TACE regimens varied (cTACE/DEB-TACE), outcomes did not differ significantly, likely owing to procedural standardization, uniform chemotherapy (doxorubicin/platinum-based), and consistent ischemic mechanisms outweighing chemotherapeutic differences. Group A demonstrated superior liver function and survival, attributable to multiple mechanisms: I. Reduced TACE sessions (2.98 vs. 5.04, p < 0.001) minimized iatrogenic injury, aligning with evidence that fewer procedures preserve hepatic function (53); II. MWA’s precision spares functional parenchyma versus repetitive TACE (54); III. MWA maintains vascular integrity, unlike TACE-induced ischemic insults (55); IV. MWA-mediated immunomodulation alleviates immunosuppression and promotes systemic anti-tumor immunity (56). In present study, a total of 43 patients had follow-up data exceeding 2 years, demonstrating a durable treatment response. Among our followed-up patients, no new immune-related adverse events emerged after 24 months, although ongoing monitoring continues. Consistent with the present findings, existing studies indicate that most immune-related AEs occur early during treatment, with only rare occurrences beyond 24 months (Kitano et al. (57); Sabaté Gallego et al. (58)).

This study has several limitations. First, this multicenter, retrospective study included cases from four participating units; however, the baseline demographics were well-matched between the two groups, and the data were accurately recorded. Second, patients with portal vein/hepatic vein tumor thrombosis in the main trunks were excluded. Third, the patients included in the study were from four participating centers within the same medical construction region, which had close technical communication, training, and homogeneity in the TACE and MWA techniques and the treatment concepts for uHCC. Therefore, this study presents combined treatment strategies that may not be suitable for other medical regions. Furthermore, the retrospective design of this study, coupled with the heterogeneity in therapeutic agents employed, may diminish the statistical robustness of the outcomes. Nevertheless, no statistically significant differences were observed in drug usage profiles between the two patient cohorts. Importantly, univariate analysis of individual agents demonstrated that the survival benefits associated with each regimen were consistent with the survival advantage. Moreover, the treatment effects remained robust across multiple clinical endpoints, including ORR, PFS, and OS. This is the first large clinical case-control study to provide evidence of combining local and system therapy to treat patients with uHCC.

In conclusion, the combination treatment mode involving TACE plus MWA combined with a TKI and ICIs demonstrated a longer PFS and OS with a manageable safety profile in patients with uHCC.

## Data Availability

The raw data supporting the conclusions of this article will be made available by the authors, without undue reservation.

## References

[1] Sung H Ferlay J Siegel RL Laversanne M Soerjomataram I Jemal A. Global cancer statistics 2020: GLOBOCAN estimates of incidence and mortality worldwide for 36 cancers in 185 countries. CA Cancer J Clin. (2021) 71:209­249. doi: 10.3322/caac.21660, PMID: 33538338

[2] Samant H Amiri HS Zibari GB. ddressing the worldwide hepatocellular carcinoma: epidemiology, prevention and management. J Gastrointest Oncol. (2021) 12:S361–73. doi: 10.21037/jgo.2020.02.08, PMID: 34422400 PMC8343080

[3] Calderaro J Seraphin TP Luedde T Simon TG. Artificial intelligence for the prevention and clinical management of hepatocellular carcinoma. J Hepatol. (2022) 76:1348–61. doi: 10.1016/j.jhep.2022.01.014, PMID: 35589255 PMC9126418

[4] Lin J Zhang H Yu H Bi X Zhang W Yin J. Epidemiological characteristics of primary liver cancer in mainland China from 2003 to 2020: A representative multicenter study. Front Oncol. (2022) 12:906778. doi: 10.3389/fonc.2022.906778, PMID: 35800051 PMC9253580

[5] Chang Y Jeong SW Young Jang J Jae Kim Y. Recent updates of transarterial chemoembolilzation in hepatocellular carcinoma. Int J Mol Sci. (2020) 21:8165. doi: 10.3390/ijms21218165, PMID: 33142892 PMC7662786

[6] Zhu P Liao W Zhang WG Chen L Shu C Zhang ZW. A prospective study using propensity score matching to compare long-term survival outcomes after robotic-assisted, laparoscopic, or open liver resection for patients with BCLC stage 0-A hepatocellular carcinoma. Ann Surg. (2023) 277:e103–11. doi: 10.1097/SLA.0000000000005380, PMID: 35081573

[7] Deng M Li SH Guo RP. Recent advances in local thermal ablation therapy for hepatocellular carcinoma. Am Surg. (2023) 89:1966–73. doi: 10.1177/00031348211054532, PMID: 34743609

[8] Chidambaranathan-Reghupaty S Fisher PB Sarkar D. Hepatocellular carcinoma (HCC): epidemiology, etiology and molecular classification. Adv Cancer Res. (2021) 149:1–61. doi: 10.1016/bs.acr.2020.10.001, PMID: 33579421 PMC8796122

[9] Izzo F Mason MC Silberfein EJ Massarweh NN Hsu C Tran Cao HS. Long-term survival and curative-intent treatment in hepatitis B or C virus-associated hepatocellular carcinoma patients diagnosed during screening. Biol (Basel). (2022) 11:1597. doi: 10.3390/biology11111597, PMID: 36358298 PMC9687526

[10] Gupta P Maralakunte M Kumar-M P Chandel K Chaluvashetty SB Bhujade H. Overall survival and local recurrence following RFA, MWA, and cryoablation of very early and early HCC: a systematic review and Bayesian network meta-analysis. Eur Radiol. (2021) 31:5400–8. doi: 10.1007/s00330-020-07610-1, PMID: 33439319

[11] Yang J Guo W Lu M. Recent perspectives on the mechanism of recurrence after ablation of hepatocellular carcinoma: A mini-review. Front Oncol. (2022) 12:895678. doi: 10.3389/fonc.2022.895678, PMID: 36081558 PMC9445307

[12] Galle PR Dufour JF Peck-Radosavljevic M Trojan J Vogel A. Systemic therapy of advanced hepatocellular carcinoma. Future Oncol. (2021) 17:1237­1251. doi: 10.2217/fon-2020-0758, PMID: 33307782

[13] Zeng H Xu Q Wang J Xu X Luo J Zhang L. The effect of anti-PD-1/PD-L1 antibodies combined with VEGF receptor tyrosine kinase inhibitors versus bevacizumab in unresectable hepatocellular carcinoma. Front Immunol. (2023) 14:1073133. doi: 10.3389/fimmu.2023.1073133, PMID: 36756114 PMC9900113

[14] Xie D Sun Q Wang X Zhou J Fan J Ren Z. Immune checkpoint inhibitor plus tyrosine kinase inhibitor for unresectable hepatocellular carcinoma in the real world. Ann Transl Med. (2021) 9:652. doi: 10.21037/atm-20-7037, PMID: 33987350 PMC8106062

[15] Griffiths CD Zhang B Tywonek K Meyers BM Serrano PE. Toxicity profiles of systemic therapies for advanced hepatocellular carcinoma: A systematic review and meta-analysis. JAMA Netw Open. (2022) 5:e2222721. doi: 10.1001/jamanetworkopen.2022.22721, PMID: 35849393 PMC9295000

[16] Zhang J Pan T Zhou W Zhang Y Xu G Xu Q. Long noncoding RNA LINC01132 enhances immunosuppression and therapy resistance via NRF1/DPP4 axis in hepatocellular carcinoma. J Exp Clin Cancer Res. (2022) 41:270. doi: 10.1186/s13046-022-02478-z, PMID: 36071454 PMC9454129

[17] Palmer DH Malagari K Kulik LM. Role of locoregional therapies in the wake of systemic therapy. J Hepatol. (2020) 72:277–87. doi: 10.1016/j.jhep.2019.09.023, PMID: 31954492

[18] Llovet JM De Baere T Kulik L Haber PK Greten TF Meyer T. Locoregional therapies in the era of molecular and immune treatments for hepatocellular carcinoma. Nat Rev Gastroenterol Hepatol. (2021) 18:293–313. doi: 10.1038/s41575-020-00395-0, PMID: 33510460

[19] Sidali S Trépo E Sutter O Nault JC. New concepts in the treatment of hepatocellular carcinoma. United Eur Gastroenterol J. (2022) 10:765–74. doi: 10.1002/ueg2.12286, PMID: 35975347 PMC9486494

[20] Fan Y Xue H Zheng H. Systemic therapy for hepatocellular carcinoma: current updates and outlook. J Hepatocell Carcinoma. (2022) 9:233–63. doi: 10.2147/JHC.S358082, PMID: 35388357 PMC8977221

[21] Dumolard L Ghelfi J Roth G Decaens T Macek Jilkova Z. Percutaneous ablation-induced immunomodulation in hepatocellular carcinoma. Int J Mol Sci. (2020) 21:4398. doi: 10.3390/ijms21124398, PMID: 32575734 PMC7352237

[22] Wang WQ Lv X Li J Li J Wang JL Yuan T. Repeat hepatectomy versus microwave ablation for solitary and small (≤3 cm) recurrent hepatocellular carcinoma with early or late recurrence: A propensity score matched study. Eur J Surg Oncol. (2023) 49:1001–8. doi: 10.1016/j.ejso.2022.12.016, PMID: 36585301

[23] Spiliotis AE Gäbelein G Holländer S Scherber PR Glanemann M Patel B. Microwave ablation compared with radiofrequency ablation for the treatment of liver cancer: a systematic review and meta-analysis. Radiol Oncol. (2021) 55:247–58. doi: 10.2478/raon-2021-0030, PMID: 34167181 PMC8366737

[24] Xu Z Xie H Zhou L Chen X Zheng S. The combination strategy of transarterial chemoembolization and radiofrequency ablation or microwave ablation against hepatocellular carcinoma. Anal Cell Pathol (Amst). (2019) 2019:8619096. doi: 10.1155/2019/8619096, PMID: 31534899 PMC6732647

[25] Li W Ni CF. Current status of the combination therapy of transarterial chemoembolization and local ablation for hepatocellular carcinoma. Abdom Radiol (NY). (2019) 44:2268–75. doi: 10.1007/s00261-019-01943-2, PMID: 31016345

[26] Liu C Li T He JT Shao H. TACE combined with microwave ablation therapy vs. TACE alone for treatment of early- and intermediate-stage hepatocellular carcinomas larger than 5 cm: a meta-analysis. Diagn Interv Radiol. (2020) 26:575–83. doi: 10.5152/dir.2020.19615, PMID: 32965220 PMC7664747

[27] Cho Y Choi JW Kwon H Kim KY Lee BC Chu HH. Transarterial chemoembolization for hepatocellular carcinoma: 2023 expert consensus-based practical recommendations of the korean liver cancer association. Korean J Radiol. (2023) 24:606–25. doi: 10.3348/kjr.2023.0385, PMID: 37404104 PMC10323412

[28] Freites-Martinez A Santana N Arias-Santiago S Viera A. Using the Common Terminology Criteria for Adverse Events (CTCAE - Version 5.0) to Evaluate the Severity of Adverse Events of Anticancer Therapies. CTCAE versión 5.0. Evaluación de la gravedad de los eventos adversos dermatológicos de las terapias antineoplásicas. Actas Dermosifiliogr (Engl Ed). (2021) 112:90–2. doi: 10.1016/j.ad.2019.05.009, PMID: 32891586

[29] Gordan JD Kennedy EB Abou-Alfa GK Beg MS Brower ST Gade TP. Systemic therapy for advanced hepatocellular carcinoma: ASCO guideline. J Clin Oncol. (2020) 38:4317–45. doi: 10.1200/JCO.20.02672, PMID: 33197225

[30] Finn RS Qin S Ikeda M Galle PR Ducreux M Kim TY. Atezolizumab plus bevacizumab in unresectable hepatocellular carcinoma. N Engl J Med. (2020) 382:1894–905. doi: 10.1056/NEJMoa1915745, PMID: 32402160

[31] Finn RS Ikeda M Zhu AX Sung MW Baron AD Kudo M. Phase ib study of lenvatinib plus pembrolizumab in patients with unresectable hepatocellular carcinoma. J Clin Oncol. (2020) 38:2960–70. doi: 10.1200/JCO.20.00808, PMID: 32716739 PMC7479760

[32] Ren Z Xu J Bai Y Xu A Cang S Du C. Sintilimab plus a bevacizumab biosimilar (IBI305) versus sorafenib in unresectable hepatocellular carcinoma (ORIENT-32): a randomised, open-label, phase 2-3 study. Lancet Oncol. (2021) 22:977–90. doi: 10.1016/S1470-2045(21)00252-7, PMID: 34143971

[33] Kudo M. A new treatment option for intermediate-stage hepatocellular carcinoma with high tumor burden: initial lenvatinib therapy with subsequent selective TACE. Liver Cancer. (2019) 8:299–311. doi: 10.1159/000502905, PMID: 31768341 PMC6872999

[34] Jin ZC Zhong BY Chen JJ Zhu HD Sun JH Yin GW. Real-world efficacy and safety of TACE plus camrelizumab and apatinib in patients with HCC (CHANCE2211): a propensity score matching study. Eur Radiol. (2023) 33:8669–81. doi: 10.1007/s00330-023-09754-2, PMID: 37368105 PMC10667391

[35] Xue J Ni H Wang F Xu K Niu M. Advances in locoregional therapy for hepatocellular carcinoma combined with immunotherapy and targeted therapy. J Interv Med. (2021) 4:105–13. doi: 10.1016/j.jimed.2021.05.002, PMID: 34805958 PMC8562181

[36] Cai M Huang W Huang J Shi W Guo Y Liang L. Transarterial chemoembolization combined with lenvatinib plus PD-1 inhibitor for advanced hepatocellular carcinoma: A retrospective cohort study. Front Immunol. (2022) 13:848387. doi: 10.3389/fimmu.2022.848387, PMID: 35300325 PMC8921060

[37] Yuan Y He W Yang Z Qiu J Huang Z Shi Y. TACE-HAIC combined with targeted therapy and immunotherapy versus TACE alone for hepatocellular carcinoma with portal vein tumour thrombus: a propensity score matching study. Int J Surg. (2023) 109:1222–30. doi: 10.1097/JS9.0000000000000256, PMID: 37026861 PMC10389515

[38] Suwa K Seki T Aoi K Yamashina M Murata M Yamashiki N. Efficacy of microwave ablation versus radiofrequency ablation for hepatocellular carcinoma: a propensity score analysis. Abdom Radiol (NY). (2021) 46:3790–7. doi: 10.1007/s00261-021-03008-9, PMID: 33675382 PMC8286931

[39] Ni Y Huang G Yang X Ye X Li X Feng Q. Microwave ablation treatment for medically inoperable stage I non-small cell lung cancers: long-term results. Eur Radiol. (2022) 32:5616–22. doi: 10.1007/s00330-022-08615-8, PMID: 35226157

[40] Zaitoun MMA Elsayed SB Zaitoun NA Soliman RK Elmokadem AH Farag AA. Combined therapy with conventional trans-arterial chemoembolization (cTACE) and microwave ablation (MWA) for hepatocellular carcinoma >3-<5 cm. Int J Hyperthermia. (2021) 38:248–56. doi: 10.1080/02656736.2021.1887941, PMID: 33615957

[41] Gordon AC Lewandowski RJ. CBCT-guided TACE-MWA for HCC Measuring up to 5 cm. Acad Radiol. (2021) 28:S71–2. doi: 10.1016/j.acra.2021.05.012, PMID: 34154903

[42] Keshavarz P Raman SS. Comparison of combined transarterial chemoembolization and ablations in patients with hepatocellular carcinoma: a systematic review and meta-analysis. Abdom Radiol (NY). (2022) 47:1009–23. doi: 10.1007/s00261-021-03368-2, PMID: 34982183

[43] Lu B Zhu L Wang X Zhong L Cheng Y Fan J. Effects of radiofrequency ablation combined with transarterial chemoembolization and antiviral therapy on the prognosis and quality of life in primary chronic HBV-related liver cancer. J Buon. (2019) 24:1979–84., PMID: 31786864

[44] Meng M Li W Yang X Huang G Wei Z Ye X. Transarterial chemoembolization, ablation, tyrosine kinase inhibitors, and immunotherapy (TATI): A novel treatment for patients with advanced hepatocellular carcinoma. J Cancer Res Ther. (2020) 16:327–34. doi: 10.4103/jcrt.JCRT_101_20, PMID: 32474520

[45] Li X Liang P Ye X. TATI modality: A new perspective on the treatment of advanced hepatocellular carcinoma. J Cancer Res Ther. (2020) 16:957–9. doi: 10.4103/jcrt.JCRT_850_20, PMID: 33004734

[46] Leuchte K Staib E Thelen M Gödel P Lechner A Zentis P. Microwave ablation enhances tumor-specific immune response in patients with hepatocellular carcinoma. Cancer Immunol Immunother. (2021) 70:893–907. doi: 10.1007/s00262-020-02734-1, PMID: 33006650 PMC7979675

[47] Xu J Zhang Y Jia R Yue C Chang L Liu R. Anti−PD−1 antibody SHR−1210 combined with TKIs for advanced hepatocellular carcinoma, gastric, or esophagogastric junction cancer: An open−label, dose escalation and expansion study. Clin Cancer Res. (2019) 25:515−23. doi: 10.1158/1078-0432.CCR-18-2484, PMID: 30348638

[48] Xu F Wei Z Ye X. Immunomodulatory effects of microwave ablation on Malignant tumors. Am J Cancer Res. (2024) 14:2714–30. doi: 10.62347/QJID8425, PMID: 39005685 PMC11236778

[49] Sang J Liu P Wang M Xu F Ma J Wei Z. Dynamic changes in the immune microenvironment in tumor-draining lymph nodes of a lewis lung cancer mouse model after microwave ablation. J Inflammation Res. (2024) 17:4175–86. doi: 10.2147/JIR.S462650, PMID: 38979433 PMC11228081

[50] Xu F Sang J Wang N Wang M Huang Y Ma J. Microwave ablation combined with immune checkpoint inhibitor enhanced the antitumor immune activation and memory in rechallenged tumor mouse model. Cancer Immunol Immunother. (2025) 74:161. doi: 10.1007/s00262-025-04003-5, PMID: 40131498 PMC11937475

[51] Wang M Sang J Xu F Wang S Liu P Ma J. Microwave ablation combined with flt3L provokes tumor-specific memory CD8+ T cells-mediated antitumor immunity in response to PD-1 blockade. Adv Sci (Weinh). (2025) 12:e2413181. doi: 10.1002/advs.202413181, PMID: 39629989 PMC11775548

[52] Patel SA Nilsson MB Le X Cascone T Jain RK Heymach JV. Molecular mechanisms and future implications of VEGF/VEGFR in cancer therapy. Clin Cancer Res. (2023) 29:30–9. doi: 10.1158/1078-0432.CCR-22-1366, PMID: 35969170 PMC10274152

[53] Li J Wang N Shi C Liu Q Song J Ye X. Short-term efficacy and safety of callispheres drug-loaded microsphere embolization in primary hepatocellular carcinoma. J Cancer Res Ther. (2021) 17:733–9. doi: 10.4103/jcrt.JCRT_1848_20, PMID: 34269307

[54] Floridi C Cacioppa LM Rossini N Macchini M Bruno A Agostini A. Microwave ablation followed by cTACE in 5-cm HCC lesions: does a single-session approach affect liver function? Radiol Med. (2024) 129:1252–64. doi: 10.1007/s11547-024-01842-7, PMID: 38958915 PMC11322225

[55] Zhu C Chen H Fang Q Jiang Y Xu H. Improvement in the condition of patients with primary liver cancer with transcatheter arterial chemoembolization before and after microwave ablation interventional therapy. Am J Transl Res. (2021) 13:11908–16., PMID: 34786121 PMC8581931

[56] Chen S Zeng X Su T Xiao H Lin M Peng Z. Combinatory local ablation and immunotherapies for hepatocellular carcinoma: Rationale, efficacy, and perspective. Front Immunol. (2022) 13:1033000. doi: 10.3389/fimmu.2022.1033000, PMID: 36505437 PMC9726793

[57] Kitano M Honda T Hikita E Masuo M Miyazaki Y Kobayashi M. Delayed immune-related adverse events in long-responders of immunotherapy: a single-center experience. Asia Pac J Clin Oncol. (2024) 20:463–71. doi: 10.1111/ajco.14059, PMID: 38608154

[58] Sabaté Gallego M Pérez Esquirol E Garcia Doladé N Vidal Guitart X Carreras Soler MJ Farriols Danés A. Incidence and characteristics of adverse drug reactions in a cohort of patients treated with PD-1/PD-L1 inhibitors in real-world practice. Front Med (Lausanne). (2022) 9:891179. doi: 10.3389/fmed.2022.891179, PMID: 36072949 PMC9441693

